# Older adults’ beliefs about coping strategies for anxiety: A UK-based multicultural qualitative study informed by Leventhal’s Common-Sense Model of Self-Regulation

**DOI:** 10.1371/journal.pone.0346995

**Published:** 2026-10-08

**Authors:** Rasha Alkholy, Penny Bee, Rebecca Pedley, Karina Lovell

**Affiliations:** 1 Division of Nursing, Midwifery and Social Work, School of Health Sciences, Faculty of Biology, Medicine and Health, The University of Manchester, Manchester, United Kingdom; 2 Manchester Academic Health Science Centre, The University of Manchester, Manchester, United Kingdom; RMIT University, AUSTRALIA

## Abstract

**Aim:**

Older adults experiencing anxiety disorders, particularly those from minority ethnic backgrounds, are less likely to use formal mental health services than younger adults. This UK-based multicultural qualitative study explored beliefs underpinning coping strategies for anxiety among White British, South Asian, African and Caribbean older adults who self-reported anxiety, informed by Leventhal’s Common-Sense Model.

**Methods:**

Individual semi-structured interviews were conducted with 52 older adults aged ≥65 who self-reported current or past anxiety. Professional interpreters supported interviews with non-English-speaking participants (n = 10). Eight public contributors collaborated on different aspects of the study. Data were managed and analysed using the Framework Method.

**Findings:**

The study drew on the perspectives of 27 participants with distressing anxiety and 25 with non-distressing anxiety. Across all cultural groups, participants adopted a range of coping strategies, with self-help strategies being the most prominent. Our findings suggest that coping strategy selection was shaped by factors extending beyond cultural background. Participants tended to adopt strategies that were congruent with their salient identities, perceived need for support, candidacy for healthcare, social relationships, and beliefs about the appropriateness and effectiveness of different responses. This complexity helps explain variation in perceptions both across and within cultural groups. Inductive analysis identified new dimensions not recognised in Leventhal’s Model: factors underlying the motivation to take action, beliefs about treatment providers and new treatment-related beliefs.

**Conclusions:**

Interventions that support older adults to apply self-help strategies more effectively may represent acceptable adjuncts to formal therapy. Nevertheless, addressing barriers to formal help-seeking remains essential, particularly among those with perceived need for professional support. No one model can depict the complexity of coping behaviours. While applying Leventhal’s Model offered novel insights, it could not fully capture the motivational factors underlying engagement with specific coping behaviours. Cross-cultural research should attend to within-group heterogeneity rather than impose assumptions of cultural homogeneity.

## 1 Introduction

Despite their negative impact on health [1] and healthcare costs [2], anxiety disorders remain largely underdiagnosed and undertreated. Older adults with anxiety disorders are less likely to use formal mental health services compared to their younger counterparts [3] – a trend that is particularly evident among those from minority ethnic backgrounds [4]. Furthermore, when older adults do seek help, they are less likely to receive treatment [5], and if they do, are more often offered psychotropic medications than psychological therapy [6].

Such disparities in mental healthcare use and treatment provision require extensive exploration if we are to achieve health equity. Unfortunately, research has predominantly overlooked factors underlying these disparities beyond sociodemographic and need factors [4]. One set of factors implicated in differential health services use in physical health problems are psychosocial factors, including people’s beliefs about illness and its treatment. These beliefs are central to Leventhal’s Common-Sense Model of Self-Regulation [7]. This model outlines how individuals conceptualise health threats, select coping actions and respond to feedback from their actions. It divides the behavioural process into three stages: a perceptual stage, in which a common-sense representation of the threat is developed (i.e., illness representations); a response stage, in which coping strategies are activated; an appraisal stage, in which coping strategies are evaluated for their success in achieving desired goals [8]. Coping strategies encompass self-help, help-seeking, taking medication or engaging in treatment and the action plans needed for individuals to implement them [9]. Similar to illness representations, beliefs about treatment (i.e., treatment representations) include the treatment’s identity (i.e., label and experiences when used), cause (i.e., mode of action), timeline (i.e., treatment duration, time to effect), consequences or adverse effects, perceived effectiveness to control or cure the illness [10] and emotional reactions [11]. A key aspect of the model is the need for coherence between an individual’s illness representation, their chosen coping strategies and their perception that their desired goals are being met [12].

In a previous analysis [13], we explored older adults’ beliefs and perceptions of anxiety (i.e., illness representations) and found that their understanding of anxiety lacked clarity – participants neither considered anxiety as a ‘normal’ part of ageing nor conceptualised it as an ‘illness’. This ambiguity may help explain the low rates of mental health service utilisation in this population. However, to fully understand why older adults do not seek help for anxiety, despite experiencing distressing symptoms, it is necessary to look beyond illness representations alone and explore the beliefs that underpin their coping strategies. A cross-cultural examination is particularly important, as cultural norms and values can influence these beliefs [14]. Such insights may help us develop more acceptable services that are responsive to the needs of culturally diverse older adult populations.

Although Leventhal’s Common-Sense Model [10] has been extensively applied in physical health conditions [9,15–17] and increasingly in mental health conditions [18–21], evidence supporting its applicability to mental health problems in older adults remains limited. Existing studies in this population have focused primarily on illness representations of dementia among people living with dementia and their caregivers [22], with little research examining common mental health problems such as anxiety and depression in later life.

Moreover, previous qualitative studies exploring illness perceptions in anxiety have focused on younger populations, including adolescents in the UK [23] and children and young people in Indonesia [24]. This underscores a significant gap in the extant literature and highlights the need for further qualitative inquiry in this area. To our knowledge, no study has yet explored older adults’ beliefs about coping strategies for anxiety using Leventhal’s Common-Sense Model [10].

## 2 Methodology and methods

### 2.1 Study design

In this paper, we present a distinct analysis of primary data from a large multicultural qualitative dataset [25] examining perceptions of anxiety and coping strategies among White British, South Asian, African and Caribbean older adults in the UK who self-reported anxiety. While a separate paper [13] reports an analysis focused specifically on older adults’ perceptions and representations of anxiety, the present paper addresses a different research question and offers a distinct conceptual contribution. Here, we explore participants’ beliefs about coping strategies through the lens of Leventhal’s Common‑Sense Model [10]. Although both papers draw on the same dataset, they employ analytically independent thematic frameworks and address non‑overlapping aspects of the data. We employed the qualitative approach described by Ritchie and Spencer [26]; a pragmatic methodology that aligns with subtle realism and interpretivism.

### 2.2 Patient and public involvement and engagement group

Eight public contributors collaborated on different aspects of the study: five White British, two South Asian and one Caribbean, all living in the UK. Most of the public contributors were 65 years or over (n = 7) and had lived experiences of common mental health problems (n = 5). They coproduced the study materials, recruitment strategy and an arts-based public engagement workshop, served as Recruitment Champions, contributed to data interpretation and the final thematic framework, and co-authored a commentary on their involvement experience [13].

### 2.3 Ethical approval

The study received ethical approval from the University of Manchester Research Ethics Committee (Ref: 2021-10461-17766; Date: 05/02/2021).

### 2.4 Sampling and recruitment

Participants were eligible if they were 65 years or over; self-identified as being “worriers”, had experienced or were experiencing anxiety or “stress”; self-identified as being White British, South Asian (Indian, Pakistani, Bangladeshi), African or Caribbean; and living in the UK. Individuals were excluded if they were receiving mental health crisis management. We used purposive sampling to ensure adequate representation across the three cultural groups and snowball sampling to maximise our reach to eligible participants.

Data were primarily collected during the national COVID-19 lockdown. We asked relevant gatekeepers (e.g., Older Black Africans Day Opportunities, the African Caribbean Care Group, Khush Amdid), research networks (e.g., Centre for Applied Dementia Studies at the University of Bradford) and our public contributors to circulate the study details to their contacts. Study flyers were posted on study-specific social media platforms (X/Twitter, Facebook), as well as on the websites and newsletters of the University of Manchester and third-sector and charity organisations (e.g., Anxiety UK, Parkinsons UK). Physical study flyers were distributed in venues like local churches, mosques and pharmacies to reach digitally excluded older adults.

Due to concerns about the acceptability of the term “anxiety” among older adults, our public contributors recommended the use of different terms in the study flyers, such as “stress” and “worrier” and their synonyms in Urdu, Hindi and Bengali.

Older adults interested in the study were encouraged to get in direct contact with the researcher using the contact details provided in the study advert. Older adults who knew about the study through gatekeepers or public contributors and explicitly stated that they would prefer to be contacted by the researcher or required interpretation services were asked for their consent to share one method of contact.

During initial contact, the researcher provided a brief verbal explanation of the study and screened potential participants for their eligibility to take part. Potential participants were told that this study aimed to understand people’s experiences of anxiety that persisted over long periods and impacted their daily functioning or caused significant distress. Those who self-reported anxiety, either currently or in the past, based on this broad description, and those who had a clinical diagnosis of anxiety were eligible to participate.

Eligible participants were provided with a ‘Research Package’ including the Participant Information Sheet, consent materials and three questionnaires. Consent materials included a Participant Reference Copy of the Consent Form, which participants could refer to while providing verbal consent, and a Documentation of Verbal Consent Form, which served as a script for the researcher during the audio-recorded verbal consent process. The questionnaires included a sociodemographic form to capture basic sociodemographic data, and the Geriatric Anxiety Scale [27] and Geriatric Depression Scale-Short Form [28] to screen for current symptoms of anxiety and depression, respectively. The study advert, Participant Information Sheet, the Geriatric Anxiety Scale [27] and the Participant Reference Copy of the Consent Form were translated into Urdu, Hindi and Bengali. All translations were proofread and verified by certified independent translators. The Urdu study materials were also reviewed by an Urdu-speaking public contributor to ensure linguistic appropriateness and cultural acceptability. The English [28], Urdu [29], Hindi [30] and Bengali [31] versions of the Geriatric Depression Scale-Short Form are available from the respective source publications. The Geriatric Anxiety Scale total scores were only used to evaluate the effectiveness of our inclusive recruitment strategy and were not used as an eligibility criterion.

Upon receipt of the study materials, participants were invited to an introductory meeting with the researcher to review the Participant Information Sheet and address questions. Professional interpreters facilitated introductory meetings with non-English-speaking participants. This was particularly important for non-English-speaking participants who communicated in languages other than Urdu, Hindi or Bengali, as additional time was required to thoroughly review and interpret the information sheet.

Participants were informed that participation was voluntary and that, even after providing consent, they retained the right to withdraw. To mitigate potential coercion, participants identified and approached by public contributors or gatekeepers were informed that their decision to participate or not would remain confidential. All participants were afforded at least 24 hours to consider their consent for participation prior to scheduling their interviews.

### 2.5 Data collection

Data collection started during the COVID-19 pandemic and spanned successive phases of national restrictions. Therefore, the approved ethics application included procedures for conducting interviews remotely (via telephone or the University Zoom account) and for obtaining audio-recorded verbal consent in place of written consent. As restrictions eased, some interviews were conducted face-to-face. However, the approved verbal consent procedure was retained and applied consistently across all participants.

Participants completed two interviews: an initial brief interview during which verbal consent and answers to the three questionnaires were audio-recorded separately, followed by a semi-structured interview lasting 38–160 minutes.

At the beginning of the interview, the researcher provided a summary of the study information, addressed any remaining questions and reminded participants of their right to decline participation or withdraw from the study at any point. After participants agreed to audio-record the consent procedure, the researcher used the Documentation of Verbal Consent Form as a script, read each consent statement aloud and asked participants to clearly state whether they agreed or disagreed. Verbal consent was audio-recorded separately from participants’ answers to the three questionnaires and was documented in a secure consent log.

No independent witness was present during the consent process. Instead, consent was verified through the audio-recording of the verbal-consent procedure alongside documentation of consent in the secure consent log. Requiring a witness was considered impractical given the remote nature of data collection. It could also have impacted participants’ willingness to take part, particularly if the witness was a family member or friend, and could have compromised confidentiality by disclosing the participant’s involvement in the study, especially if a gatekeeper acted as the witness.

Verbal consent audio files contained identifiable information and were stored separately from the research data on a restricted-access, password-protected University of Manchester research data storage service. These recordings were retained as essential study files for five years in accordance with the University of Manchester’s research data retention policy and were not made available as part of data sharing.

Audio-recorded verbal consent procedures and the associated documentation were reviewed and approved by the University of Manchester Research Ethics Committee (Ref: 2021-10461-17766; Date: 05/02/2021). This approach complied with institutional guidance for conducting research during the COVID-19 pandemic and ensured a secure, auditable record of informed consent.

The semi-structured interviews followed a topic guide, coproduced with the public contributors**,** based on Leventhal’s Common-Sense Model [10]. The topic guide has been reported in detail elsewhere [13] and is provided as supplementary material (S1 File). The guide included five open-ended questions with follow-up probes to explore participants’ conceptualisation of anxiety, their self-help strategies, help-seeking behaviours and beliefs about treatments, perceived barriers and enablers to mental health services use, and the outcomes they used to appraise different strategies. We iteratively adapted the topic guide to include new topics or perspectives introduced by the participants. In this paper, we report only the findings related to coping strategies (i.e., self-help strategies, help-seeking behaviours, beliefs about treatments, barriers and enablers to service use, appraisal outcomes).

Ten interviews were conducted in non-English languages, including Wolof (n = 1), Luganda (n = 2), Urdu (n = 4), Punjabi (n = 2) and Sylheti (n = 1). Twelve interviews were conducted in person (seven at participants’ homes, three in community organisations, two in university facilities), 27 by telephone and 13 virtually (University Zoom account).

Interviews were audio recorded using encrypted devices, transcribed verbatim in English and anonymised. Transcripts were verified against recordings for accuracy. Data collection took place in the UK over a 13-month period (23 April 2021 to 30 May 2022). All participants received a £30 gift voucher in recognition of their time.

### 2.6 Data analysis

We used the Framework Method [26] to manage and analyse the data. This comparative form of thematic analysis involves using an organising framework of deductively and inductively derived themes as a scaffold against which data are analysed. Its hallmark feature is its matrix output consisting of rows (cases), columns (codes) and cells of ‘summarised data’, providing a structure for systematically reducing and analysing the data both by case and code. The matrix output allows a bird’s-eye view of the entire dataset while maintaining the integrity of each participant’s account [32], a feature particularly relevant to our study as we wanted to compare participants’ perspectives both across and within the three cultural groups. Leventhal’s Common-Sense Model [7] served as the a priori organising framework that guided the initial deductive analysis. We also used inductive analysis to extend and refine the thematic framework, allowing the identification of new dimensions not recognised by the original model.

RA familiarised herself with the data by reviewing the audio-recordings and transcripts (stage 1: familiarisation). She then identified initial codes and developed a preliminary thematic framework, using a combined inductive-deductive approach (stage 2: developing the initial thematic framework), which was subsequently refined and systematically applied across the whole dataset (stage 3: indexing/sorting). Indexed data were charted into framework matrices (stage 4: charting) to facilitate both within-case and cross-case comparisons. This process enabled the identification of patterns in the data, the development of typologies and the generation of explanatory insights and linkages (stage 5: mapping/interpretation).

The research team (KL, PB, RP, RA) met regularly throughout this analytical process to discuss and verify coding, refine the thematic framework and review matrix outputs and interpretations. Public contributors reviewed selected extracts of anonymised quotes and provided input on data interpretation and the final thematic framework. Any discrepancies were resolved to ensure consensus among the wider group. Data management was supported by NVivo 12 Plus.

We agreed that data saturation was reached when codes, themes, linkages and interpretations were judged to be sufficiently developed, conceptually dense and coherent to address the study’s objectives. Data saturation was reached after interviews with 14 White British, 16 South Asian and 15 African or Caribbean participants. To validate this assessment, we conducted one to two additional interviews with the African/Caribbean and White British groups, respectively. A further four interviews were conducted with the South Asian group to ensure adequate linguistic diversity within the sample.

The interviewer (RA) is a former physician with training in qualitative research and had no prior relationship with any participant. Any safety concerns that arose during data collection were discussed with the lead clinician (KL). RA used reflexive notes to document assumptions, decisions and dynamics that may have impacted the research process, highlighting personal, interpersonal, methodological and contextual issues. These reflections were discussed iteratively with the research team. An audit trail was used to record all research steps.

## 3 Findings

### 3.1 Participant characteristics

We interviewed 52 older adults: 16 White British, 20 South Asian and 16 African or Caribbean. The mean age of the participants was 73 years, and most of them were females.

Although all participants self-reported current or past experiences of anxiety, our sample comprised two distinct subgroups: those who, at the time of experiencing anxiety, experienced significant distress (defined as a subjective sense of emotional pain accompanied by an inability to cope) and/or functional impairment (defined as limitations in important areas of functioning attributable to anxiety) and those who did not. We refer to the first group as participants ‘with distressing anxiety’ and the latter as participants ‘with non-distressing anxiety’. This study draws on the perspectives of 27 older adults with distressing anxiety and 25 with non-distressing anxiety. The characteristics of the participants are outlined in Table 1.

**Table 1:** Participant characteristics.

Participant characteristic	White British (n = 16)	South Asian (n = 20)	African/Caribbean (n = 16)	Total (n = 52)
**Age (years), mean (SD)**	73.6 (6.6)	70.9 (6.6)	75.4 (8.4)	73.1 (7.3)
**Age range (years)**	66 - 91	65 - 86	66 - 88	65 - 91
**Sex**				
Female, n (%)	10 (62.5)	14 (70.0)	13 (81.3)	37 (71.2)
Male, n (%)	6 (37.5)	6 (30.0)	3 (18.7)	15 (28.2)
**Education (years), mean (SD)**	14.4 (3.7)	12.9 (6.8)	13.7 (4.5)	13.6 (5.3)
**Marital status**				
Single	0	0	2	2
Married	10	11	4	25
Separated	0	0	3	3
Divorced	3	2	3	8
Widowed	2	7	4	13
Prefer not to say	1	0	0	1
**Language used**				
English	16	13	13	42
Non-English	0	7	3	10
• Urdu	0	4	0	4
• Punjabi	0	2	0	2
• Sylheti	0	1	0	1
• Luganda	0	0	2	2
• Wolof	0	0	1	1
**Living status**				
Living alone	4	4	9	17
Living with others	12	16	7	35
**Needs help in performing daily activities**				
Always	2	3	0	5
Most of the time	1	3	1	5
Sometimes	2	2	1	5
Rarely	2	2	2	6
Never	9	10	12	31
**Medical problems**				
Yes	16	18	13	47
No	0	2	3	5
**Participants with distressing anxiety**	**9**	**10**	**8**	**27**
Currently experiencing symptoms suggestive of anxiety based on GAS total score^A^				
• Yes	8	9	6	23
• No	1	1	2	4
Currently experiencing symptoms of depression based on GDS-SF score^B^				
• No depression	2	3	6	11
• Suggestive of depression	5	2	2	9
• Almost always depression	2	5	0	7
Sought help from healthcare services for mental health concerns^C^				
• Yes	9	6	6	21
• No	0	4	2	6
Has a clinically diagnosed mental health condition (i.e., formal diagnosis by a healthcare professional)				
• None	4	6	7	17
• Anxiety	1	1	0	2
• Depression	1	1	0	2
• Anxiety and other comorbidities (depression, post-traumatic stress disorder)	3	2	1	6
Received treatment for anxiety (irrespective of formal diagnosis)				
• Medication	6	4	3	13
• Psychotherapy	5	1	4	10
**Participants with non-distressing anxiety**	**7**	**10**	**8**	**25**
Currently experiencing symptoms suggestive of anxiety based on GAS total score^A^				
• Yes	6	3	2	11
• No	1	7	6	14
Currently experiencing symptoms of depression based on total GDS-SF score^B^				
• No depression	7	10	8	25
Sought help from healthcare services for mental health concerns^C^				
• Yes	2	3	2	7
• No	5	7	6	18
Has a clinically diagnosed mental health condition (i.e., formal diagnosis by a healthcare professional)				
• None	7	8	7	22
• Anxiety and other comorbidities (depression)	0	0	1	1
• Other mental health problems (bipolar disorder, obsessive compulsive disorder)	0	2	0	2
Received treatment for anxiety (irrespective of formal diagnosis)				
• Medication	0	0	0	0
• Psychotherapy	1	0	0	1

GAS = Geriatric Anxiety Scale; GDS-SF = Geriatric Depression Scale-Short Form; n = number; SD = standard deviation.

^A^Geriatric Anxiety Scale total score: > 9 currently experiencing symptoms suggestive of anxiety; ≤ 9 currently not experiencing symptoms suggestive of anxiety [33].

^B^Geriatric Depression Scale-Short Form total score: ≤ 5 no depression; 6–10 suggestive of depression; ≥ 11 almost always depression [28].

^C^ Any contact with formal healthcare services, including primary, secondary or tertiary services, and private or public services, for mental health reasons.

### 3.2 Thematic overview

We developed a thematic framework (Fig 1) incorporating a priori themes derived from Leventhal’s Common-Sense Model [10], against which data were deductively analysed, and potential new themes identified via inductive thematic analysis. These new themes include factors underlying the motivation to take action (or barriers and enablers to help-seeking) and beliefs about treatment providers. Beliefs about medications and psychotherapy were generally aligned with the a priori treatment-related dimensions proposed by Leventhal’s Common-Sense Model [10]. Additional new treatment-related beliefs were identified: conflicts with higher-order goals, beliefs about compensatory behaviours and factors which may determine the effectiveness of therapy.

**Fig 1:**
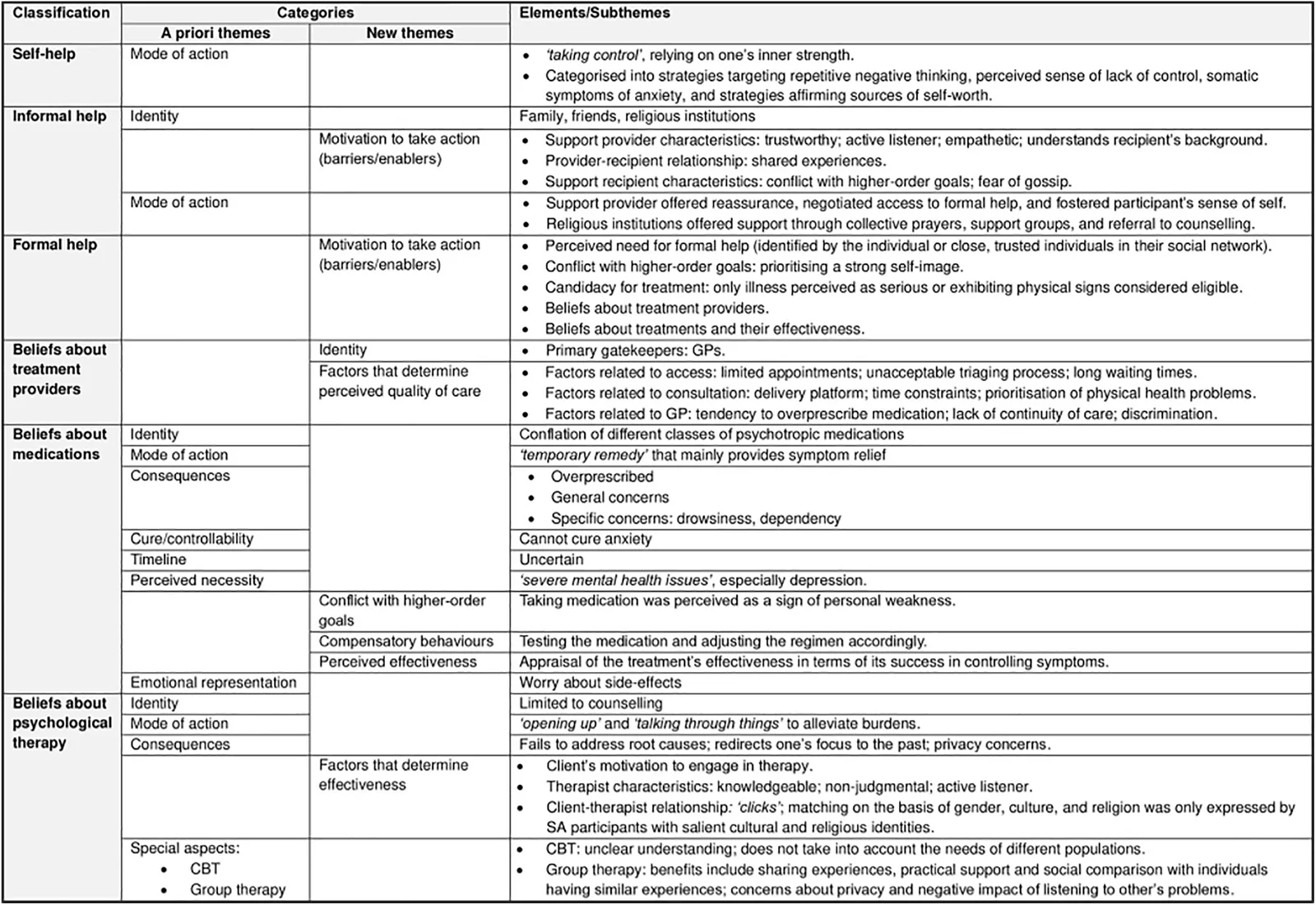
Thematic framework demonstrating older adults’ beliefs about coping strategies for anxiety.

#### 3.2.1 Self-help.

Across all cultural groups, participants emphasised the importance of *‘taking control’* and relying on their inner strength to manage their mental health concerns before seeking any form of support. *‘Self-help’* represented a toolkit of strategies used by individuals as a first-line, and sometimes the only, approach to coping with mental health concerns.

*‘It’s kind of, you know, taking steps yourself…Anything that comes out of your own is much better than any intervention by an external person, you know.’ (South Asian male; non-distressing anxiety, used self-help only)*

Self-help strategies were categorised according to their primary target into strategies targeting repetitive negative thinking patterns like worry and rumination, somatic symptoms of anxiety, perceived sense of lack of control, and strategies affirming sources of self-worth.

***Strategies targeting repetitive negative thinking***. Distraction was the most frequently cited self-help strategy across all cultural groups. Participants engaged in activities such as walking, watching TV and exercising, so that they were *‘not sitting in the house and dwelling on things.’* These activities helped them shift their focus from negative thoughts to less taxing ones. However, participants noted that the effects of such strategies were typically short-lived.

***Strategies targeting symptoms of anxiety.*** These were mainly behavioural strategies used by participants who attributed somatic symptoms, such as hyperventilation and gastrointestinal disturbance, to anxiety. They included breathing exercises and drinking soothing, warm fluids.

***Strategies targeting a perceived sense of lack of control.*** Participants believed their anxiety was caused by unmodifiable stressors beyond their control, including progressive illness, cumulative social losses and traumatic or discriminatory experiences. This lack of control over one’s life was difficult to accept, especially among those with distressing anxiety.

*‘Things you can’t logically, you can’t control. I can control what I do today, I can control what I do tomorrow. But you can’t change the past, can you? But I couldn’t seem to accept this, and I’d dwell on it.’ (White British male; distressing anxiety, sought formal help, but refused treatment)*

Participants with salient religious identities, mainly from the African/Caribbean and South Asian groups, were able to resign their desire to be in control to a higher power who would *‘not forsake*’ them, but rather guide and protect them. Religious prayers offered a chance to connect and converse with God, thus providing comfort and reassurance.

*‘But when you start a day and you pray and you commit everything to him [God], you go out and you have, it’s almost like you have an authority, you have a confidence, I’ve handed over everything to him and now he is in control and I am just going to be listening for God’s guidance.’ (African/Caribbean female; distressing anxiety, used self-help only)*

***Strategies affirming sources of self-worth.*** Across all cultural groups, participants employed diverse strategies that shared a common underlying goal of maintaining an overarching positive view of the self. These strategies include self-affirming reflections, engagement in meaningful and purposeful activities, downward social comparison and involvement in social groups, particularly those one strongly identifies with. When participants perceived an unmodifiable stressor (e.g., progressive illness) as a threat to a specific aspect of their self-concept (i.e., independence), they leaned on and reinforced other unaffected aspects of their self (i.e., personal strengths and achievements, relationships and group memberships).

African/Caribbean participants tended to use self-affirming reflections as a reminder of their diverse and strong personal qualities instead of focusing on threatened aspects of their identity.

*‘You have to tell yourself, “I am great, I am a wise woman. Yes, I am a grandmother, I am a professional, these are all roles, but they are not who I am. I am a wise woman.” Write it on the wall, say it to yourself, it is conditioning.’ (African/Caribbean female; distressing anxiety, sought formal help, but refused treatment)*

Participants from all cultural groups engaged in various activities, such as sewing, gardening and exercise, with the main goal of distracting themselves. However, only those participants who set achievable goals and focused on outcomes felt a sense of accomplishment that boosted their self-esteem when they achieved those outcomes.

*‘…that [gardening] was really great for my mental health because I was nurturing these plants, oh so proud, and potatoes, I grew potatoes as well.’ (African/Caribbean female; distressing anxiety, sought formal help, received counselling and medication)*

Participants tended to seek comparisons with those who were worse off to feel better about themselves. However, for those with salient religious identities, downward social comparison seemed to be an inherent part of their belief.

*‘Because when you listen to others it will just help you with your situation. If you are a Christian, a Muslim or somebody you believe in God, you will just go back to the comfort of your house and just give thanks to God.’ (African/Caribbean male; non-distressing anxiety, used self-help only)*

Participants also leaned on their relationships and group memberships to buffer the effect of threats to other aspects of their identity. Across all cultural groups, participants tended to join social groups based on age, activity (e.g., singing), disease (e.g., diabetes) or social roles (carers groups) to make friends, engage in activities and obtain practical advice. Only one participant was aware of social prescribing services, linking people to community organisations, but did not use them.

Although these groups offered social support, it was group memberships founded on shared, salient identities that fostered a sense of belonging, purpose and meaning. This pattern was evident among South Asian and African/Caribbean participants with salient religious and cultural identities, both of which were strongly *‘interlinked’.* African/Caribbean participants regarded in-group members who shared their religious beliefs and cultural background as an extension of their self. For these participants, the Church was not only a place of worship; it was a family.

*‘I know the church and the pastor…you become one family, all the members are sisters and brothers, yeah.’ (African/Caribbean female; distressing anxiety, sought formal help, received counselling)*

South Asian women with salient religious or cultural identities explained how joining South Asian women-only social groups allowed them to connect with women who shared their cultural traditions, religious beliefs and language while respecting deeply rooted cultural norms and expectations.

*‘Yes, that’s the reason that [name of social group] came, isn’t it? You bringing them [South Asian women] out from their understanding and, you know, mixing with people, that’s how important going out is how important, how helpful for your mental health is seeing when, because especially Asian community our community, women are left alone, isn’t it?’ (South Asian female; non-distressing anxiety, used self-help only)*

#### 3.2.2 Informal support (i.e., family, friends, religious institutions) for mental health concerns.

Family and friends represented valuable social networks that participants turned to when experiencing mental health concerns. However, only a select few within these networks were trusted to provide support. These individuals understood the participant’s background and could relate to what they were going through because of their shared experiences, *‘we’re all in the same boat’.* They offered reassurance, encouragement and practical support when needed and, in some cases, negotiated the participant’s access to formal help. These people *‘give you strength’* - they fostered the participants’ sense of value.

*‘They [grandchildren] understand very much, yes, and they even tell me, they told me, “We lost our grandpa, now we don’t want to lose you, so you have to be strong and conquer.” (African/Caribbean female; distressing anxiety, received informal help)*

Talking in itself had a cathartic effect.

*‘If I didn’t tell anyone then it will damage, you know, I’m thinking, thinking, thinking so much and my heart, I’m not happy. When I tell someone like my sister or my friend then after that I’m okay, that’s okay.’ (South Asian female; distressing anxiety, sought formal help, received medication)*

Participants sought support from friends rather than close family members when they felt *‘it’s probably easier to talk to somebody outside’,* especially if they did not want to *‘burden’* their families or felt their families were *‘dismissive’.*

However, not all participants sought support from family or friends. Across all cultural groups, participants who valued maintaining a *‘strong’* persona were *‘embarrassed’* about seeking support. Similarly, those who prioritised projecting a *‘positive’* image avoided seeking help because they believed that people who were *‘always moaning about something’* were shunned by others.

*‘No, no, I don’t talk to many people about my feelings because it’s always people ringing me up to ask me for advice, most of my friends and, you know…cause I’m like the matriarch of the family, and everybody would come to me, as a bigger sister here.’ (African/Caribbean female; distressing anxiety, sought formal help, received medication)*

*‘You see, there’s one lady who sits with us at the club and nothing’s ever right for her, and you realise that if you’re like that, people say, “Oh, I don’t want her sitting next to me,” do you know what I mean? Because she talks negative.’ (White British female; non-distressing anxiety, used self-help only)*

South Asian and African/Caribbean participants tended to be especially concerned about gossip.

*‘Interpreter: …they [her friends] won’t keep it a secret and then they’ll just gossip about her.’ (South Asian female; distressing anxiety, used self-help only)*

African/Caribbean participants with salient religious identities relied heavily on their religious institutions for mental health support. Besides the power of collective prayers, participants with distressing anxiety appreciated the opportunity to be referred to a *‘trained therapist’* when needed and to join support groups established by their local Church to receive and offer reciprocal support from others with similar experiences.

*‘In our area, we have those ‘home-help’, now if we live in the same community, you come together, you fellowship together. So, in that process…I state my problem, then I am told, you know, there is somebody else like this, so when you meet all together, you come to see maybe you share the same problem or their problem is worse than yours, or me, I can encourage them a bit, how I used to go to counselling or something like that.’ (African/Caribbean female; distressing anxiety, sought informal help, received counselling)*

#### 3.2.3 Formal help (i.e., healthcare providers) for mental health concerns.

Participants’ accounts suggest that formal help-seeking was not a straightforward response to anxiety. Instead, decisions to seek professional support were shaped by the interplay between perceived need for support, judgements of candidacy or legitimacy for healthcare, identity-related concerns, and beliefs about treatments and treatment providers. These considerations shifted throughout participants’ illness journeys. Formal help-seeking was more likely when perceived need outweighed concerns about candidacy, identity and treatment.

This section focuses on perceived need, candidacy for healthcare and identity-related concerns. Beliefs about treatment providers and specific treatments are considered in the following section.

Perceived need for support was conceptualised as a personal threshold at which functional impairment and emotional distress resulting from anxiety surpassed the participant’s perceived ability to cope. Some participants believed this threshold was only reached if anxiety was associated with ‘*erratic’* or harmful behaviours, such as risk to self or others and alcohol or substance abuse.

Perceived need was not always recognised by participants themselves. Close, trusted individuals often played a significant role in identifying need for support. They either encouraged or discouraged participants from seeking help.

*‘…my elder daughter, she had said, “Dad, I think it would really help you to talk to someone.” (African/Caribbean male; distressing anxiety, sought formal help, received counselling)*

However, perceiving need for support does not necessarily translate into formal help-seeking. Participants’ accounts suggest that perceived need was weighed against judgements about candidacy for professional support. These judgements were closely linked to participants’ broader conceptualisations of illness, types of illness and their perceived consequences. Participants tended to view professional support as legitimate primarily for health conditions characterised by visible, objective signs or serious consequences. Therefore, less visible conditions, such as chronic pain and mental health conditions, were not commonly regarded as appropriate candidates for professional support.

*‘For some time, I’d been working myself up to see a GP and I thought, well, you know, what’s he going to think of me? But no, he was, I thought he’d be such, sort of, ‘What’s he worried about when I’ve got people here with terminal illnesses?’ But no, he was glad I came, and we had quite a long chat.’ (White British male; distressing anxiety, sought formal help, but refused treatment)*

In comparison to anxiety, depression was more commonly perceived as a clearer and more legitimate reason for seeking professional support. Participants with distressing anxiety who experienced comorbid depression often described seeking formal help primarily for depression rather than anxiety. Our findings suggest that anxiety occupies an ambiguous conceptual position, whereby it may be experienced as distressing and causing functional impairment, yet not recognised as a legitimate reason for seeking professional support.

This ambiguity may help explain why participants with distressing anxiety who had sought formal help commonly described substantial delays in help-seeking, often spanning years. They described the period leading to help-seeking as one of severe distress, using terms such as *‘felt absolutely awful’, ‘desperation’*, *‘miserable’*, *‘suicidal’* and *‘crisis’.*

*‘I suppose it was a bit of a crisis. I just said to myself, I don’t want to carry on living this way, permanently afraid, permanently upset…I said to myself, ‘This isn’t life, life should not be like this, it can’t be like this, I’ve got to do something about it.’ (White British male; distressing anxiety, sought formal help, but refused treatment)*

Help-seeking also had implications for self-image. Participants who prioritised projecting a *‘strong’* self-image and interpreted help-seeking for mental health concerns ‘*as a sign of weakness*’, delayed or refused to seek help. This was particularly evident among male participants for whom seeking help conflicted with expectations of masculinity.

*‘We (men) should be seen as being the strongest one, you know.’ (White British male; distressing anxiety, sought formal help, received medication and counselling)*

#### 3.2.4 Beliefs about treatment providers.

Across all cultural groups, most participants considered general practitioners (GPs) the primary gatekeepers to formal mental healthcare services. However, their views varied concerning the role of GPs in treating anxiety and mental health problems in general. Some believed GPs were well-equipped to manage common mental health problems, while others contended that their role should be limited to referring people to *‘trained therapists’*, psychologists or psychiatrists. Conversely, participants who had chronic physical health conditions, such as Parkinson’s Disease, expressed the importance of specialist nurses and, to a lesser extent, specialist doctors, in the early detection of mental health problems and subsequent referral to appropriate health services.

Across all cultural groups, participants’ evaluations of the quality of healthcare provided by their GP were polarised, with the majority inclining towards a negative view. Participants who did not seek help for mental health concerns often had preconceived ideas about GPs’ propensity to overprescribe antidepressants as a *‘quick fix’* for mental health concerns rather than investigate more complex underlying issues. Consequently, this discouraged them from seeking help.

Those who sought formal help explained how access to GP appointments was limited during the COVID-19 lockdown. This was further complicated by an unacceptable triaging process conducted by non-medical personnel, *‘how can a* *receptionist triage a patient? It’s beyond my ken.’* These barriers forced participants to seek other sources of support.

Even when offered a GP consultation, long waiting times and sudden transition to phone consultations were considered unacceptable. Time constraints during GP consultations meant that participants had to prioritise physical health problems over their mental health concerns. Furthermore, participants who were not consistently seen by the same GP often perceived GPs as dismissive. This lack of continuity of care was viewed as a significant barrier to appreciating the person’s narrative and taking account of their unique experiences and needs.

*‘There’s one thing, every time you go to the GP there’s a different doctor. If it’s one that okay, they know your background, but if every time new face, different one.’ (South Asian female; distressing anxiety, sought formal help, but refused treatment)*

Some South Asian and African/Caribbean participants reported experiencing discrimination in healthcare settings based on their cultural background, which was frequently associated with implicit assumptions regarding their socioeconomic status. Participants reported that racial stereotyping and lack of compassion, understanding and respect by GPs adversely affected the quality of care they received. In some cases, the substandard care and distress associated with these discriminatory experiences aggravated the participants’ mental health concerns.

*‘…some GPs will treat someone from a minority community less respectfully than maybe someone from the White community because the assumption made is, you know, in some socioeconomic wards of [name of city] where we know literacy rates are poor, [South Asian] men or women, when they go to the GP, or it could be a Middle Eastern lady or man struggling with English will be dismissed by the GP, you know, because they don’t know the background.’ (South Asian male; non-distressing anxiety, used self-help only)*

African/Caribbean participants noted that society’s disregard for older adults was not limited to general social interactions, but extended into healthcare settings. They feared that this would negatively impact the quality of healthcare services they would receive in the future.

*‘But I don’t want to be, when I’m old be classed, I am old, right, but to be put with elderly, to be segregated.’ (African/Caribbean female; distressing anxiety, sought formal help, received medication and counselling)*

However, participants’ views about GPs were not unanimously negative. A limited number of participants believed their GP, particularly when consistent, helped them understand their symptoms and provided a diagnosis that allowed them access to different services.

#### 3.2.5 Beliefs about treatments.

***Beliefs about medications.*** Across all cultural groups, participants conflated different classes of psychotropic medications, including antidepressants, benzodiazepines, antipsychotic medications and beta-blockers. Most participants believed medication should be reserved for those with *‘severe mental health issues’*, especially depression. This perspective was particularly relevant when depression was attributed to *‘more of a chemical thing than a mental thing’*.

A limited subset of participants believed that medication could be used for anxiety, but only as a *‘last resort’* if it causes prolonged significant distress, functional impairment or is complicated by other serious symptoms, such as psychosis. Participants with distressing anxiety who prioritised the projection of a strong personal identity tended to regard the use of medications for anxiety as a sign of personal weakness. Among them, those who required medication grappled with the conflict between their need for medication and how they interpreted this need.

*‘I used to be very strong and I had strong willpower, you know, I can manage. I have so many problems in my life, but I can manage really. But, when my husband passed away, after that I am getting weak and weak and weak, psychologically and emotionally and physically. Then this COVID came and suddenly, I don’t know where from, you know. So obviously I think I want to try more but I can’t. I think maybe need ((pause)) but I don’t know how to cope with it [anxiety], you know, without medicine.’ (South Asian female; distressing anxiety, sought formal help, received medication and counselling)*

Medication was generally depicted as *‘a temporary remedy’* for anxiety that mainly provides symptom relief, but *‘doesn’t solve the problem’.* Nevertheless, participants across all cultural groups had a predominantly negative view of psychotropic medications, expressing general, *‘Any drugs you take, they all have a side-effect’*, as well as specific concerns. The most feared side effects were drowsiness and dependence. Medications were believed to *‘slow you down’* and make you *‘like a bit of a zombie’.* Participants highlighted their fear of becoming ‘*dependent’* on *‘the happy tablets’,* which they perceived as *‘addictive’*. This perception was often reinforced by the notion of withdrawal symptoms following the abrupt cessation of medication. Such beliefs served as barriers to accepting medications as viable treatment options, even among those who were currently taking them. Other side effects included drug-drug interactions, especially in the context of polypharmacy, drug-food interactions, fatigue, dry mouth and gastrointestinal disturbance.

Participants evaluated the perceived necessity of medications and their potential benefits against possible adverse effects, including their impact on the self, to determine whether to initiate medication. To overcome their concerns, some participants took their prescribed medication irregularly, used lower than the recommended doses or asked their GP to avoid the *‘stronger’* medications. Essentially, they were testing the medications, weighing their effectiveness in controlling symptoms against side effects to determine what they considered to be the optimal dose [34]. Those who passively accepted medication tended to be older adults with distressing anxiety and comorbid depression. Participants were generally uncertain about when the medications were expected to take effect and how long they should be taken.

***Beliefs about psychological therapies.*** Participants tended to limit talking therapies to counselling. Those who preferred counselling over other treatments believed that *‘opening up’* to a therapist about their problems, revisiting traumatic experiences and having the opportunity *‘to talk through things’* could help alleviate their burdens. Participants expected therapists, as professionals, to guide them through their recovery journey and empower them rather than offer predetermined solutions. Therapists were *‘detached’* from family and social circles, which facilitated the process of disclosure.

*‘You lighten yourself to strangers you don’t know.’ (South Asian female; distressing anxiety, sought formal help, received medication and counselling)*

Conversely, participants who criticised counselling argued that it fails to address the root causes of mental health concerns and instead redirects one’s focus to the past rather than the future. These participants were also sceptical of sharing private problems with *‘strangers’*, preferring to keep their problems to themselves or discuss them with trusted family members, friends or, in the case of the African/Caribbean group, their *‘pastor’.*

*‘Probably, I feel it’s [the therapist] a stranger, I know they’re professional people, but I feel that it’s a stranger, I’m going to talk about my personal life, I’d rather talk to who I’m close to, like I said my mother, my big sister, them the people I feel they’ll understand.’ (South Asian male; distressing anxiety, sought formal help, received medication)*

The effectiveness of talking therapies, in general, was believed to depend on the individual’s motivation to engage in therapy*, ‘If the person doesn’t want to help himself, nobody can help’*, and the therapist being a knowledgeable, supportive, non-judgmental, active listener who could *‘click’* or *‘gel’* with the client. The importance of client-therapist matching on the basis of gender, culture and religion was only expressed by South Asian participants with salient cultural and religious identities.

Only White British participants with distressing anxiety recalled that they had received cognitive behavioural therapy (CBT). Nevertheless, participants from all cultural groups had an unclear understanding of CBT. Those who did not receive CBT tended to confuse it with counselling, while those who received CBT considered it a *‘tool to use’* that should be frequently administered and struggled to remember what it involved. CBT was believed to be an intervention that does not take into account the needs of different populations.

*‘So, we have a huge push by organisations or medical professionals to promote CBT as a main line of intervention. It may work for, again I hate to use the word, it might work for a ‘middle class White professional’, but sad to say, it probably will not work as good as an intervention for people whose English language is poor, have low literacy levels or skills.’ (South Asian male; non-distressing anxiety, used self-help only)*

Group therapy was welcomed by participants who believed it could offer an opportunity to share experiences, gain practical advice and feel that they are not alone. However, it was perceived as unacceptable by those who were concerned about privacy and the negative impact listening to other people’s problems could have on their mental health.

## 4 Discussion

### 4.1 Summary of the findings

This UK-based multicultural qualitative study explored beliefs underlying coping strategies among White British, South Asian, African and Caribbean older adults who self-reported anxiety, and examined the applicability of Leventhal’s Common-Sense Model [10] within this context. Participants adopted a range of coping strategies, including self-help, informal support and formal help-seeking, which shifted throughout their illness journey. Self-help strategies were fundamental to all participants and were perceived as most meaningful when they drew on salient personal and social (i.e., cultural, religious) identities, thus reinforcing a positive sense of self and self-worth. Informal support was perceived as acceptable and meaningful when it was congruent with participants’ salient identities (e.g., desired self-image or religious identity), embedded within trusted, non-judgemental social networks and shaped by shared experiences. Decisions to seek formal help reflected the interplay between perceived need for support, perceived candidacy for healthcare, competing identity-related goals, and beliefs about treatments and treatment providers.

Taken together, our findings suggest that the selection of coping strategies was shaped by factors extending beyond cultural background. Older adults tended to adopt strategies that were congruent with their salient identities, perceived need for support, perceived candidacy for healthcare, social relationships, and beliefs about the appropriateness and effectiveness of different responses. This complexity helps explain variation in perceptions both across and within cultural groups.

Through inductive analysis, we identified additional dimensions not recognised in Leventhal’s Model: factors underlying the motivation to take action, beliefs about treatment providers and new treatment-related beliefs.

### 4.2 Self-help

Our findings suggest that older adults predominantly relied on self-help strategies to manage anxiety, consistent with previous research on depression [35–39] and loneliness [40] in later life. However, our study extends this literature by showing how self-help strategies were not experienced as equally beneficial. Participants differentiated between strategies that provided temporary relief from distress and those that were perceived as more meaningful as they drew on valued self-resources, including salient personal and social identities.

By leveraging these self-resources, participants were able to shift their focus away from their vulnerabilities towards distinct, unaffected, positive and valued aspects of themselves. This enabled them to broaden their self-view and restore an overarching positive self-image [41]. Our findings suggest that the value of self-help can extend beyond symptom reduction to include the affirmation of identity and self-worth.

These findings highlight the potential value of approaches that attend to human capacity rather than human deficiency [42]. Interventions that can help older adults identify and draw on their existing strengths may represent acceptable adjuncts or, in mild cases, alternatives to formal therapeutic approaches. One such group of interventions is social prescribing services that connect individuals to non-clinical sources of support, including participation in social groups [43]. Participants’ accounts suggest that meaningful, goal-directed activities and group involvement served functions that extended beyond mere pleasure. Such activities provided older adults with avenues to affirm positive and desirable aspects of their identity [44]. Moreover, older adults who strongly identified with their groups gained access to additional social and psychological resources – valuable social networks, a sense of self-esteem, belonging, meaning and purpose. This aligns with the extensive body of “social cure” research, which suggests that internalised, positive group memberships act as social cures promoting well-being and health [45]. This was particularly evident among South Asian and African/Caribbean participants who had salient religious and cultural identities and joined groups characterised by these interlinked, harmonious identities.

### 4.3 Informal support

Our findings suggest that older adults do not universally seek informal support, even when it is available. Instead, participants sought such support selectively, perceiving it as acceptable and meaningful when it was congruent with their salient identities (e.g., salient religious identity), embedded within trusted, non-judgemental social networks and shaped by shared experiences. These findings may help explain why the social support literature has consistently shown a weak correlation between received and perceived support [46], a subconstruct of social support that has been consistently linked with positive health outcomes [47,48]. Our study extends this literature by showing how provider characteristics (i.e., trustworthy, active listener, empathetic, understands recipient’s background), recipient characteristics (i.e., prioritising competing identity-related goals) and the provider-recipient relationship (i.e., shared experiences) shape whether received support is perceived as acceptable and meaningful.

The significance of shared experiences also resonates with qualitative evidence [4] showing that older adults often rely on others with shared experiences of anxiety and depression as potential sources of informal support. Shared experiences may validate the individual’s own experiences and enhance the credibility and relevance of advice and support offered.

### 4.4 Formal help-seeking

Our findings suggest that older adults weighed their perceived need for support against their perceived candidacy for healthcare, competing identity-related goals, and concerns about treatments and treatment providers when deciding whether to seek formal help for anxiety. These considerations appeared to shift across participants’ illness journeys.

Perceived need for support appeared to operate as a personal threshold, reached when functional impairment and emotional distress resulting from anxiety surpassed older adults’ subjective (or objective, as observed by others) ability to cope. This is consistent with a substantial body of evidence that identifies perceived need as a strong predictor of mental health service use [4], and potentially a more appropriate construct for defining treatment gaps than categorical diagnostic criteria alone [49]. However, reaching this threshold did not necessarily translate into formal help-seeking.

Participants’ accounts suggest that perceived need was weighed against judgements about candidacy or legitimacy for healthcare. Identification of candidacy, as conceptualised by Dixon-Woods and colleagues [50], refers to individuals’ recognition of their symptoms as legitimate grounds for medical attention. This process is shaped by broader cultural understandings of illness. Such understandings have been shown to shape differential experiences of healthcare, for example between patients with cancer and heart failure [51]. In our study, older adults tended to view health conditions characterised by visible, objective signs or serious consequences as more legitimate reasons for professional support than anxiety, thus reflecting broader assumptions that ‘less visible’ forms of distress may be less deserving of medical attention.

This failure to consider anxiety as a legitimate reason for seeking formal help, despite experiencing distressing anxiety, may reflect older adults’ ambiguous understanding of anxiety. This ambiguity was particularly evident in comparison with depression, which was more readily recognised as warranting professional support. Previous qualitative research [13] suggests that older adults experiencing anxiety were often left in limbo – neither perceiving anxiety as a normal emotional response nor recognising it as an illness warranting professional support. In contrast, depression was more commonly understood as a serious condition requiring treatment.

The decision to seek formal help was further complicated by competing identity-related goals. Consistent with previous evidence [4,52], participants’ accounts indicate that help-seeking was often delayed or avoided when it was perceived as threatening valued identities, such as being strong or positive.

Finally, beliefs about treatments and treatment providers also shaped older adults’ help-seeking behaviour. Some participants were reluctant to seek formal help because they anticipated that anxiety would be treated primarily with medications, which they viewed as a *‘quick fix’* for problems rooted in social suffering and not amenable to medical treatment. Moreover, participants had a predominantly negative view of psychotropic medications because of concerns about dependency, addiction and general side effects. These findings echo the literature on beliefs about medicines for chronic illness [34,53] which suggests that people weigh perceived need for medications against concerns when deciding whether to take medication and adhere to it.

Similar considerations influenced attitudes towards psychological therapies. Decisions to seek or engage with psychological therapies were shaped by expectations of benefits and harms of therapy, past experiences, the availability of informal sources of support and beliefs about factors required for therapy to be effective. However, in line with previous research [54], participants often demonstrated an unclear understanding of psychological therapies, primarily limiting them to counselling.

Participants’ perceptions of treatment providers also influenced help-seeking. Although general practitioners are considered the primary gatekeepers to formal mental healthcare services within the National Health Service (NHS), our findings suggest that concerns regarding access to appointments, features of the consultation itself and the quality of the general practitioner’s interpersonal skills often discouraged help-seeking. In particular, our findings highlight the significance of continuity of care in fostering trusting and respectful provider-patient relationships that facilitate the disclosure of mental health concerns and help-seeking.

Notably, some South Asian and African/Caribbean participants described experiences of discrimination in healthcare settings that they attributed to their cultural background and associated with implicit assumptions about their socioeconomic status. These findings are concerning given growing evidence that implicit stereotypes held by physicians can compromise the patient-provider relationship [55] and contribute to inequities in healthcare [56]. Hence, our findings lend support to growing calls for greater cultural humility; a process of “committing to an ongoing relationship with patients, communities, and colleagues” that requires “humility as individuals continually engage in self-reflection and self-critique” ([57], p. 118).

### 4.5 Leventhal’s Common-Sense Model

Overall, our findings support the applicability of Leventhal’s Common-Sense Model in understanding how older adults select coping strategies for anxiety. However, they also suggest that the transition from health threat appraisal to enacting coping behaviours (e.g., formal help-seeking) is not as straightforward as the model may imply. Instead, this process appears to involve judgements about identity congruence, perceived need for support, perceived candidacy for healthcare, the quality of social relationships, and beliefs about the appropriateness and effectiveness of different responses.

Help-seeking was sometimes perceived as a threat to valued self-identities, rather than as a source of support. A basic premise of Leventhal’s Common-Sense Model [58] is that individuals are motivated to select and perform coping behaviours that reduce or control a health threat (i.e., not experiencing anxiety). While the model recognises the importance of the self-system and identity-related goals in self-regulation, it does not fully capture how multiple, potentially competing goals can shape help-seeking decisions. Indeed, goals are embedded within complex hierarchies, where more abstract, identity-related goals are pursued through more concrete, behavioural goals. Moreover, at any one time, individuals have multiple goals that differ in importance, that may either facilitate or interfere with one another [59]. Conflicts between goals may arise due to the mutual incompatibility of the behaviours needed to attain them [60]. Our findings suggest that help-seeking was sometimes appraised as a potential threat to valued identities. Prioritising a strong or positive self-image often precluded participants from seeking help.

Understanding these identity-related tensions requires recognition that older adults draw on multiple salient identities, including personal, role and social identities, when evaluating coping strategies. These identities are dynamic and contextually defined; they interact and intersect in complex ways. Across all cultural groups, older adults tended to adopt coping strategies that were congruent with their salient identities. This finding suggests that coping decisions are influenced by efforts to maintain identity coherence. It also highlights the limitations of conceptualising culture as a relatively static force that divides people into distinct, internally homogeneous groups and portrays individuals as passive recipients, devoid of individual agency [61]. Such approaches risk neglecting within-group differences by disregarding the crucial role of one’s multifaceted self-identity in interpreting self-relevant events, selectively adopting cultural patterns and regulating behaviour. Future cross-cultural research should embrace the diversity and heterogeneity of individuals both within and across groups rather than homogenise entire cultural groups.

### 4.6 Strengths and limitations

This study is the first multicultural qualitative inquiry to explore older adults’ beliefs about coping strategies for anxiety. A significant limitation is the recruitment of a self-reporting sample and the use of varied terms to describe anxiety. Our public contributors expressed concern that relying solely on Western medical terminology, such as “anxiety”, might imply that only individuals who had been clinically diagnosed or those who had previously sought help from healthcare services were eligible, thereby excluding potentially eligible older adults with undiagnosed anxiety. This group is particularly important, as little research has explored their perceptions of anxiety and the beliefs underpinning their coping strategies.

Public contributors who self-identified as South Asian or Caribbean also noted that medical labels may not be widely recognisable in their communities, may not translate easily into other languages, and may carry stigmatising connotations. Therefore, the public contributors and research team agreed to include lay terms such as “worrier” and “stress” in the study flyers. While this enhanced inclusivity, it also meant that participants with a wide range of experiences could have been included, potentially introducing variability in the data. Despite implementing several measures to ensure all participants had experienced anxiety either in the past or at the time of the study, we believe that the sample included two groups of participants: those who likely met the diagnostic criteria for anxiety disorders (i.e., distressing anxiety) and those who did not (i.e., non-distressing anxiety). To reflect these differences, we developed a typology distinguishing between the two groups.

## 5 Conclusions

Interventions that empower older adults to capitalise on their self-resources and deploy self-help strategies more effectively may represent acceptable adjuncts to formal therapy. Nevertheless, it is essential to address barriers that preclude older adults from seeking formal help, particularly those who perceive a need for professional support. Although no one model can depict the complexity of coping behaviours, applying Leventhal’s Common-Sense Model yielded novel insights into how older adults respond to anxiety. However, the model could not fully capture the motivational factors underlying one’s decision to seek help. Future cross-cultural research should attend to within-group heterogeneity rather than impose assumptions of cultural homogeneity if it is to generate nuanced and accurate insights.

## Supporting information

S1 File Interview topic guide. (DOCX)
