# Supplementary material for: Older adults’ beliefs about coping strategies for anxiety: A UK-based multicultural qualitative study informed by Leventhal’s Common-Sense Model of Self-Regulation
Source: PLoS One. 2026 Oct 8;21(10):e0346995. doi: 10.1371/journal.pone.0346995 (PMC13648998; doi:10.1371/journal.pone.0346995)
Supplement: Supplementary file 1 [file pone.0346995.s001.docx]

S1: Interview topic guide

| **Appointment details** |  |
| --- | --- |
| **Participant Identification Number (PIN)** |  |
| **Date** |  |
| **Time** |  |
| **Interview type** | ☐Phone |
|  | ☐Zoom |
| **Language used during the interview** |  |
| **If the interview is to be conducted in a non-English language, please complete the following:** |  |
| - **Language used** |  |
| - **Name of the interpreting services supplier used** |  |
| - **Did the linguist give verbal consent to audio-record this interview?** | YES / NO |

| 1. **Experience and understanding of anxiety** | |
| --- | --- |
| ***Objective*** | To explore illness beliefs of anxiety from the perspective of community- dwelling older adults with anxiety. |
| ***Probes/***  ***Prompts*** | - **Explore participant’s own experience and understanding of anxiety:** - Word(s) used to describe anxiety/words used by others - Emotions or feelings - Thoughts - Symptoms (bodily symptoms) - Duration, timeline - Severity - Causes - Effect on life (consequences) |
| 2. **Coping strategies** | |
| ***Objective*** | To explore older adults’ choice of coping strategies in response to anxiety. |
| ***Probes/***  ***Prompts*** | - **Choice of coping strategies:** - Reasons for choosing that strategy - Perceived effectiveness |
| 3. **Help-seeking behaviour** | |
| ***Objective*** | To explore older adults’ help seeking behaviour in response to anxiety. |
| ***Probes/***  ***Prompts*** | - **Previous experience of seeking advice from formal mental health services (e.g. GP, psychologist, etc.):** - From whom - Strategy used to manage anxiety - Perceived effectiveness - **If the participant has not previously sought advice, explore their views about asking for advice from formal mental health services:** - From whom - Views about when (and why) it is appropriate to ask for advice - **Talking about ‘anxiety’ to your GP:** - Difference in the way anxiety is described to GP compared to others, and why |
| 4. **Barriers and facilitators to mental health services utilisation** | |
| ***Objective*** | To explore barriers and facilitators to mental health services utilisation from the perspective of community-dwelling older adults with anxiety. |
| ***Probes/***  ***Prompts*** | - **Factors that help encourage participants to use mental health services** - Views about facilitators - **Factors that act as a deterrent to participants using mental health services** - Views about barriers - **Awareness of different strategies available for managing anxiety** - Different strategies - Personal experience - Views about effectiveness - Views about advantages and disadvantages - Preference of a strategy over the other and why |
| 5. **Outcome(s) of interest** | |
| ***Objective*** | To explore outcome(s) of interest, from the perspective of community- dwelling older adults with anxiety, that are used to appraise coping strategies for anxiety. |
| ***Probes/***  ***Prompts*** | - **Outcome(s) of interest used to appraise the success of management** |
| **Closing** | |
|  | Any other issues that the participant would like to raise |
