# Supplementary material for: Older adults’ beliefs about coping strategies for anxiety: A UK-based multicultural qualitative study informed by Leventhal’s Common-Sense Model of Self-Regulation
Source: PLoS One. 2026 Oct 8;21(10):e0346995. doi: 10.1371/journal.pone.0346995 (PMC13648998; doi:10.1371/journal.pone.0346995)
Supplement: Supplementary file 2 [file pone.0346995.s002.docx]

**PONE-D-26-14669**

**Older adults’ beliefs about coping strategies for anxiety: A UK-based multicultural qualitative study informed by Leventhal’s Common-Sense Model of Self-Regulation**

We would like to thank the Editor and Reviewer for their careful reading of our manuscript and for their constructive and insightful feedback. We greatly appreciate the time and effort invested in providing this feedback, which has helped us strengthen the manuscript substantially.

All revisions are shown in the tracked-changes version of the manuscript, labelled ‘Revised Manuscript with Track Changes.’ We have also submitted a clean version of the revised manuscript, labelled ‘Manuscript.’

All page and line numbers refer to the clean version of the revised manuscript labelled ‘Manuscript’.

# Summary of major revisions

In response to the Editor’s and Reviewer’s comments, we made the following revisions:

1. Expanded the ‘Introduction’ section to clarify the evidence-base for Leventhal’s Common-Sense Model in older adults with mental health conditions and to identify the specific gap addressed by this study.
2. Added the final interview topic guide as a supplementary file (S1 File) and cited it in the ‘Data Collection’ section.
3. Expanded the ‘Methods’ section to describe how verbal consent was audio-recorded, documented, stored and approved by the University of Manchester Research Ethics Committee.
4. Expanded the ‘Data Analysis’ section to describe the five stages of the Framework Method and to explain why frequency analysis was not undertaken.
5. Reorganised the ‘Formal help-seeking’ theme to improve the clarity and overall readability of the findings presented in this section.
6. Revised and reorganised the ‘Discussion’ section around the key coping strategies identified by participants, and strengthened engagement with relevant empirical and theoretical literature.
7. Revised the ‘Data Availability’ statement to clarify the ethical restrictions on publicly sharing the qualitative interview data and the process for requesting access.

Below, we provide a point-by-point response to each comment.

## Editor comment 1:

***The reviewer raised concerns that need attention. They request further grounding of the finding within the existing literature.***

**Response:**

We have addressed this point in detail in our response to **Reviewer comment 4**. In brief, we have substantially revised the ‘Discussion’ section to situate our findings more clearly within relevant empirical and theoretical literature. The revised ‘Discussion’ is now organised around the main coping strategies identified in the analysis, enabling a clearer synthesis of our findings with existing evidence on self-help, informal support, formal help-seeking, treatment beliefs and treatment providers.

These revisions are reported in the revised ‘Discussion’ section (pages 35-44).

## Editor comment 2:

***They would also like more information on the Leventhal’s Common Sense Model use in older adults.***

**Response:**

We have addressed this point in detail in our response to ***Reviewer comment 1*** below. In brief, we have expanded the ‘Introduction’ section to clarify the broader evidence-base supporting the applicability of Leventhal’s Common-Sense Model across physical and mental health conditions. We also clarify that evidence concerning its application to mental health problems in older adults remains scarce, particularly in relation to anxiety. This revision helps define the gap addressed by the present study and clarifies its contribution to the literature.

These revisions are reported in the ‘Introduction’ section (pages 5-6, lines 98-108).

## Editor comment 3:

***We note that the authors’ used a topic guide based on the Leventhal’s Common-Sense Model. Please provide a copy of the final version of the topic guide/facilitation guide used by researchers.***

**Response:**

We have added the final version of the topic guide as a supplementary file (S1 File) and have cited this file in the Data Collection section. We have also noted that the topic guide has been reported elsewhere [1].

This revision is reported in the ‘Data Collection’ section (pages 11-12, lines 239-241) and the topic guide is provided as S1 File.

## Editor comment 4:

***Please ensure that your manuscript meets PLOS ONE's style requirements, including those for file naming.***

**Response:**

We have revised the author affiliations, manuscript headings, subheadings, table and supporting information citation and file naming to align with PLOS ONE style requirements. The topic guide is now provided and cited as S1 File.

These changes have been applied throughout the manuscript and supporting file.

## Editor comment 5:

***In the ethics statement in the Methods, you have specified that verbal consent was obtained. Please provide additional details regarding how this consent was documented and witnessed, and state whether this was approved by the IRB.***

**Response:**

We have revised the ‘Methods’ section to provide further details regarding the verbal consent procedure, including how consent was documented, stored and approved by the University of Manchester Research Ethics Committee (Ref: 2021-10461-17766; Date: 05/02/2021).

Specifically, we now clarify that, consent materials sent to participants as part of the ‘Research Package’ included a Participant Reference Copy of the Consent Form, which participants could refer to while providing verbal consent, and a Documentation of Verbal Consent Form, which served as a script for the researcher during the audio-recorded verbal consent process.

Before the interview, the researcher reviewed the study information with participants, addressed any remaining questions and reminded participants of their right to decline participation or withdraw at any time. After participants agreed to audio-record the consent procedure, the researcher used the Documentation of Verbal Consent Form as a script, read each consent statement aloud and recorded participants’ verbal agreement or disagreement. Verbal consent was audio-recorded separately from questionnaire responses and documented in a secure consent log.

We also clarify that no independent witness was present during the consent process. Instead, consent was verified through the audio-recording of the verbal-consent procedure alongside documentation of consent in the secure consent log. This approach was adopted because data collection was conducted remotely (because of the COVID-19 pandemic restrictions) and the inclusion of a witness was considered impractical and potentially problematic for participants’ willingness to participate and confidentiality.

Finally, we now explicitly state that the audio-recorded verbal consent procedure and associated documentation were reviewed and approved by the University of Manchester Research Ethics Committee (Ref: 2021-10461-17766; approved 05/02/2021). This approach complied with institutional guidance for conducting research during the COVID-19 pandemic and ensured a secure, auditable record of informed consent.

These revisions are reported in the ‘Methods’ section (page 9, lines 171-174; page 10, lines 201-207; pages 10-11, lines 211-237).

## Editor comment 6:

***We note that you have indicated that there are restrictions to data sharing for this study. For studies involving human research participant data or other sensitive data, we encourage authors to share de-identified or anonymized data. However, when data cannot be publicly shared for ethical reasons, we allow authors to make their data sets available upon request.***

***a) If there are ethical or legal restrictions on sharing a de-identified data set, please explain them in detail (e.g., data contain potentially identifying or sensitive patient information, data are owned by a third-party organization, etc.) and who has imposed them (e.g., a Research Ethics Committee or Institutional Review Board, etc.). Please also provide contact information for a data access committee, ethics committee, or other institutional body to which data requests may be sent.***

**Response:**

The data underlying this study comprise anonymised qualitative interview transcripts. These data cannot be made publicly available because participants consented to restricted, rather than unrestricted public, data sharing. Although anonymised, the transcripts contain rich data collected within defined demographic and community contexts, which may increase the risk of participant re-identification if shared openly. Accordingly, public access is restricted in line with the Participant Information Sheet, participant consent materials, and the ethical approval granted by the University of Manchester Research Ethics Committee (Ref: 2021-10461-17766).

The de-identified minimal dataset underlying the findings reported in this article is available to qualified researchers upon reasonable request. Requests should be submitted to the University of Manchester Research Ethics Committee ([research.ethics@manchester.ac.uk](mailto:research.ethics@manchester.ac.uk)), with the project data curator ([Rasha.Alkholy@manchester.ac.uk](mailto:Rasha.Alkholy@manchester.ac.uk)) copied into the correspondence. Requestors will be required to provide evidence of institutional affiliation and appropriate ethical approval for secondary data analysis.

A metadata record describing the anonymised dataset and the conditions for access is available on Figshare: <https://doi.org/10.48420/28468466>

We have updated the Data Availability statement in the submission form accordingly.

## Reviewer comment 1:

***Introduction. Are there any reviews or meta-analyses that have examined the role of Leventhal’s Common-Sense Model in older adults?***

We did not identify any reviews or meta-analyses that specifically examined the application of Leventhal’s Common-Sense Model in older adults with mental health conditions. We agree with the Reviewer that the previous version of the manuscript did not provide sufficient detail on this point. Therefore, we have expanded the ‘Introduction’ to clarify the broader evidence-base supporting the applicability of the model across physical and mental health conditions, while also emphasising that evidence relating to mental health conditions in older adults remains scarce. Existing studies in older adult populations have focused primarily on illness representations of dementia among people living with dementia and their caregivers, with little research examining common mental health problems such as anxiety and depression in later life.

We also note that previous qualitative studies of illness perceptions in anxiety have focused on younger populations, including adolescents in the UK and children and young people in Indonesia. This further highlights the gap addressed by the present study.

These revisions are reported in the ‘Introduction’ section (pages 5-6, lines 98-108).

## Reviewer comment 2:

***A relevant contribution that the authors may consider citing is Balsamo et al., 2015. The mediating role of early maladaptive schemas in the relation between co-rumination and depression in young adults. PloS one, 10(10), e0140177, which addresses related cognitive mechanisms. Including this reference could enhance the completeness of the literature review.***

We thank the Reviewer for this suggestion. We carefully reviewed the recommended manuscript. Balsamo et al. (2015) provides valuable insights into the role of early maladaptive schemas in the relationship between co-rumination and depression among young adults. However, we have not cited this article as it addresses a different population (young adults), a different mental health problem (depression), and a different set of cognitive mechanisms (co-rumination and maladaptive schemas). Therefore, we concluded that it was not sufficiently aligned with the aims and theoretical focus of the present study, which examines older adults’ beliefs about coping strategies for anxiety using Leventhal’s Common-Sense Model.

In response to the Reviewer’s broader point regarding the completeness of the literature review, we have expanded the ‘Introduction’ section to include additional evidence on the application of Leventhal’s Common-Sense Model in mental health and older adult populations (see above, Reviewer comment 1 for more details). We have also revised the ‘Discussion’ section to integrate our findings with relevant empirical and theoretical literature (see below, Reviewer comment 4 for more details).

These revisions are reported in the ‘Introduction’ (pages 5-6, lines 98-108) and revised ‘Discussion’ (pages 35-44) sections.

## Reviewer comment 3:

***Method. The verbal frameworks could be grouped into categories and subsequently analyzed through frequency analysis.***

**Response:**

We thank the Reviewer for this suggestion. We agree that organising participants’ accounts into analytic categories is central to qualitative analysis, and this was undertaken through our application of the Framework Method. Figure 1 in the manuscript presents the thematic framework, illustrating the main categories and themes generated from the data.

We recognise that frequency analysis can be valuable in some qualitative and mixed-methods studies. However, we did not undertake frequency analysis in the present study as it was not aligned with the interpretative focus of the study, its exploratory qualitative aim and purposive sampling strategy. Our aim was not to estimate the prevalence of particular beliefs, but to examine the range and diversity of participants’ experiences and to understand how participants themselves interpreted and attributed meaning to those experiences.

Our approach is consistent with guidance on the Framework Method, which emphasises understanding the diversity of perspectives surrounding a phenomenon rather than quantifying qualitative data [2]. Similarly, methodological guidance on interpretative analysis emphasises that *‘keyness’* of a theme is not necessarily determined by its frequency, but rather by whether it captures an important aspect of the phenomenon under study [3].

In response to this comment, we have expanded the ‘Data Analysis’ section to describe more clearly how the five stages of the Framework Method were applied: familiarisation, development of an initial thematic framework through inductive and deductive coding, indexing, charting into matrices and mapping and interpretation.

These revisions are reported in the ‘Data Analysis’ section (page 13, lines 272-280).

## Reviewer comment 4:

***Discussion. The authors should strengthen the connection between their findings and the existing literature.***

We thank the Reviewer for this important suggestion. We agree that the previous version of the manuscript would benefit from stronger engagement with the existing literature and we have revised the manuscript accordingly.

We have restructured the ‘Discussion’ section using subheadings that correspond to the main coping strategies identified in our analysis. Within each subsection, we now connect our findings with relevant literature on self-management and self-help in older adults, informal support, formal help-seeking, beliefs about medications and psychological therapies and the role of treatment providers in shaping coping decisions.

We have also expanded the final theoretical section of the ‘Discussion’ to explain how our findings support the applicability of Leventhal’s Common-Sense Model in understanding how older adults select coping strategies for anxiety. Simultaneously, we clarify that the transition from health threat appraisal to coping behaviour appears to be shaped by additional processes not fully captured in the original model.

These revisions more clearly ground our findings in existing evidence and clarify the contribution of our study to understanding the use and potential refinement of Leventhal’s Common-Sense Model.

The revised ‘Discussion’ section appears on page 35-44.

## Additional changes:

In addition to the revisions made in direct response to the Editor and Reviewer comments, we have:

- We previously stated the following in the ‘Abstract’ and ‘Summary of findings’ sections:

*“Help-seeking behaviour was influenced by a complex interplay of factors not recognised by Leventhal’s Common-Sense Model. Notably, older adults’ salient identities, rather than their cultural backgrounds, influenced their selection of coping strategies.”*

We recognised that this wording could be interpreted as suggesting that salient identities operate independently of cultural background and represent the main determinant of coping strategy selection. This was not our intention and does not accurately reflect our findings. Instead, our findings suggest that older adults’ selection of coping strategies was shaped by a complex interplay of influences extending beyond cultural background alone, including salient identities, perceived need for support, candidacy for healthcare, social relationships, and beliefs about the appropriateness and effectiveness of different responses.

Therefore, the text has been revised as follows:

*“Our findings suggest that coping strategy selection was shaped by factors extending beyond cultural background. Participants tended to adopt strategies that were congruent with their salient identities, perceived need for support, candidacy for healthcare, social relationships, and beliefs about the appropriateness and effectiveness of different responses.”*

This revision more accurately reflects the complexity of participants’ accounts and the variation observed both across and within cultural groups.

- Revised the ‘Abstract ‘to ensure that it remains within the 300-word limit;
- Reorganised the ‘Formal help-seeking’ theme to improve the clarity and overall readability of the findings presented in this section.
- Removed the funding statement at the end of the ‘Acknowledgements’ section.

We are grateful to the Editor and Reviewers for their valuable comments and hope that the revisions satisfactorily address all comments.

**References**

1.	Alkholy R, Lovell K, Pedley R, Bee P. Older adults’ beliefs about anxiety: A multicultural qualitative study informed by Leventhal’s Common-Sense Model of Self-Regulation. medRxiv. 2026:2026.03.13.26348137. doi: 10.64898/2026.03.13.26348137.

2.	Gale NK, Heath G, Cameron E, Rashid S, Redwood S. Using the Framework Method for the analysis of qualitative data in multi-disciplinary health research. BMC Med Res Methodol. 2013;13. doi: 10.1186/1471-2288-13-117.

3.	Braun V, Clarke V. Using thematic analysis in psychology. Qualitative Research in Psychology. 2006;3(2):77-101. doi: 10.1191/1478088706qp063oa.
